# Preliminary application of multiple parameters spectral CT in the diagnosis of ovarian cancer

**DOI:** 10.1097/MD.0000000000007786

**Published:** 2017-08-11

**Authors:** Tao Pang, Zhao-di Liu, Kai Deng, Cheng-qi Zhang, Guang-li Wang

**Affiliations:** CT-MR Division, Qianfoshan Hospital Affiliated to Shandong University, Jinan, Shandong, P.R. China.

**Keywords:** diagnosis, multiple parameter, ovarian cancer, spectral CT

## Abstract

The aim of the study was to evaluate the effectiveness of spectral CT in the diagnosis of ovarian cancer. We retrospectively analyzed the data of 22 patients with spectral CT enhanced scan. The patients were divided into 2 groups: ovarian cancer group (n = 11) and benign tumor group (n = 11), according to the pathologic results. CT values at 40 keV, iodine concentration (IC), water concentration (WC) and spectral curve slope (λHU) of arterial phase and venous phase in the tumors of 2 groups were measured with gemstone spectral imaging (GSI) post-processing software. The independent samples t test was used to compare these multiple parameters above between 2 groups. For the parameters which showed statistically different, the ROC curves were further generated to calculate their diagnostic effectiveness respectively. According to the results, CT values at 40 keV, IC and λHU measured in arterial and venous phases were higher in ovarian cancer group than those in benign tumor group. There were significant differences between these 2 groups (*P* < 0.05). While WC had no significant difference in these 2 groups (*P* > 0.05). CT values at 40 keV, IC and λHU had high effectiveness to the diagnosis of ovarian cancer according to ROC curves. The optimal parameter among them was IC in arterial phase with AUC of 0.90. Using 10.92 (100 ug/cm3) as a threshold value, the sensitivity and specificity for diagnosis were 88.9% and 94.7%. Thus, we concluded that spectral CT with multiple parameters was valuable in the diagnosis of ovarian cancer.

## 1 Introduction

Ovarian cancer is one of the common tumors for women. This cancer is the most lethal among the pelvic cancers, and cancer-associated mortality is as high as for cervical cancer and uterine cancer combined.^[1]^ One of the reasons is its polymorphism. Because the embryonic origin, histologic type, anatomical structure, and endocrine function of the ovary are very complex, various tumors can occur with most types of tumors in the whole body. So the early diagnosis of ovarian cancer is relatively difficult in a clinic. Moreover, more than 60% of the patients have already presented with metastatic spread beyond the pelvic when they are initially diagnosed. Recently, the advent of spectral computed tomography (CT) with gemstone spectral imaging (GSI) technique enables differentiating an iodine substance from other materials by the material decomposition principle.^[2]^ Multiple parameters can be obtained to assist diagnosis, including CT values at different energy, iodine concentration (IC), water concentration (WC), and effective atomic number. It breaks though the shortcoming of single parameter in routine CT. The multiple parameters imaging can contribute to the location, qualitative diagnosis, and grading for tumors. The aim of this study was to evaluate the effectiveness of spectral CT with multiple parameters in the diagnosis of ovarian cancer.

## 2 Materials and methods

### 2.1 Patients data

This study was a retrospective study. The protocol was approved by our institutional review board, and informed consents were obtained from all the patients.

From June 2015 to September 2016, 22 patients who revealed suspected ovarian tumors underwent contrast-enhanced CT examination using spectral CT. The age ranged from 19 to 69 years old, with the mean of 51.25 ± 7.38 years. Surgical resection was proceeded within 3 days after the CT examination. All of the results were proved by pathology. The final results were divided into 2 groups: ovarian cancer group (n = 11) and benign tumor group (n = 11). The ovarian cancers contained 3 serous adenocarcinomas, 3 mucinous adenocarcinomas, 3 Serous-mucus mixed cystadenocarcinomas, and 2 metastatic tumors. The benign tumor group included 2 ovarian fibromas, 3 serous cystadenomas, 5 mucinous cystadenomas, and 1 sclerosing stromal tumor.

### 2.2 CT protocol

An unenhanced scan followed by a dual-phase contrast-enhanced CT scan of the pelvic was performed in all the patients using spectral CT (GE Discovery CT750 HD; GE Healthcare, Milwaukee, WI) in GSI mode. The patient should take moderate contrast medium orally to make the intestine full in 4 to 6 hours before the examination. The GSI scan parameters included helical scan, dual kVp (80 and 140 kVp) momentary switched (0.5 ms), adaptive current, a detector coverage of 40 mm, a scan range from spina iliace to inferior margin of pubic symphysis, a section thickness of 5 mm, a reconstruction interval of 5 mm, a pitch of 1.375, and a gantry rotation speed of 0.5 seconds. The nonionic contrast media (Omnipaque 300 gI/100 mL, GE Healthcare, USA) at the dose of 1.2 mL/kg was injected with power injector at a rate of 3.0 mL/s through the median cubital vein. This was followed by 20 mL saline flushing at a rate of 3.0 mL/s. The arterial and venous phases were obtained at 25 to 30 seconds and 60 to 75 seconds after the injection of the contrast medium.

### 2.3 Image postprocessing

The monochromatic images with the slice thickness of 1.25 mm and spacing of 1.25 mm in the arterial and venous phases were transferred to an AW4.4 workstation (GE Volume Share 4 AW 4.4; GE Healthcare) for the analysis using a special GSI viewer. Iodine-based images, water-based images, and 101 sets of monochromatic images were obtained first. Then the CT values at 40 keV, IC, WC in the region of interest (ROI) (the relative homogeneous solid area) of the mass were measured with GSI postprocessing software. Two radiologists with more than 5 years of experience in urogenital radiology evaluated the images, respectively. In cases of discordant interpretations, decisions were reached by consensus. Each lesion was measured 3 times, and the average value would be available. The spectral curve slope (λHU) was calculated by using the formula: λHU = (CT value at 40 keV − CT value at 100 keV)/60.

### 2.4 Statistical analysis

Statistical analysis was performed using SPSS version 19.0 (IBM Corporation, Armonk, NY). The measured results were expressed as mean ± SD. The independent samples *t* test was used to compare these multiple parameters above between 2 groups. For the parameters that showed statistically different, the receiver operator characteristic (ROC) curves were further generated and area under ROC curve (AUC) was calculated to evaluate their diagnostic effectiveness. The optimal diagnostic threshold value was identified as the critical value that corresponded to the maximum of (sensitivity + specificity − 1). *P* < .05 was considered to indicate a statistically significant difference.

## 3 Results

### 3.1 Comparison of various parameters of spectral CT in 2 groups

**Figure 1:**
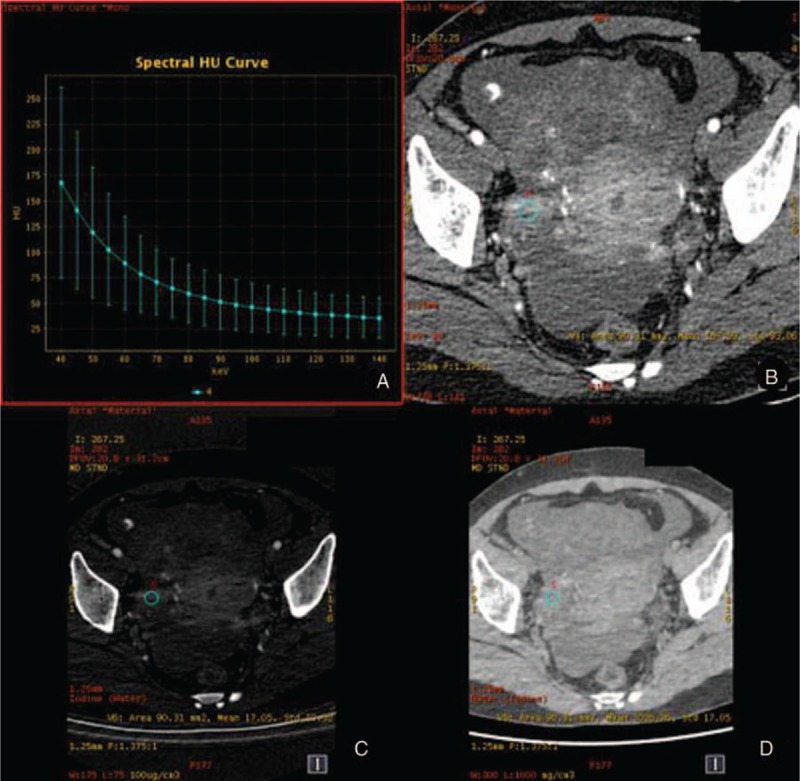
The spectral images of ovarian cancer (serous papillary adenocarcinoma) in arterial phase. A, λ_HU_ = 2.03. B, CT values at 40 keV = 167.59 HU. C, IC 17.05 (100 ug/cm^3^). D, WC 1026.26 mg/cm^3^. λ_HU_ = spectral curve slope, CT = computed tomography, IC = iodine concentration, WC = water concentration.

**Figure 2:**
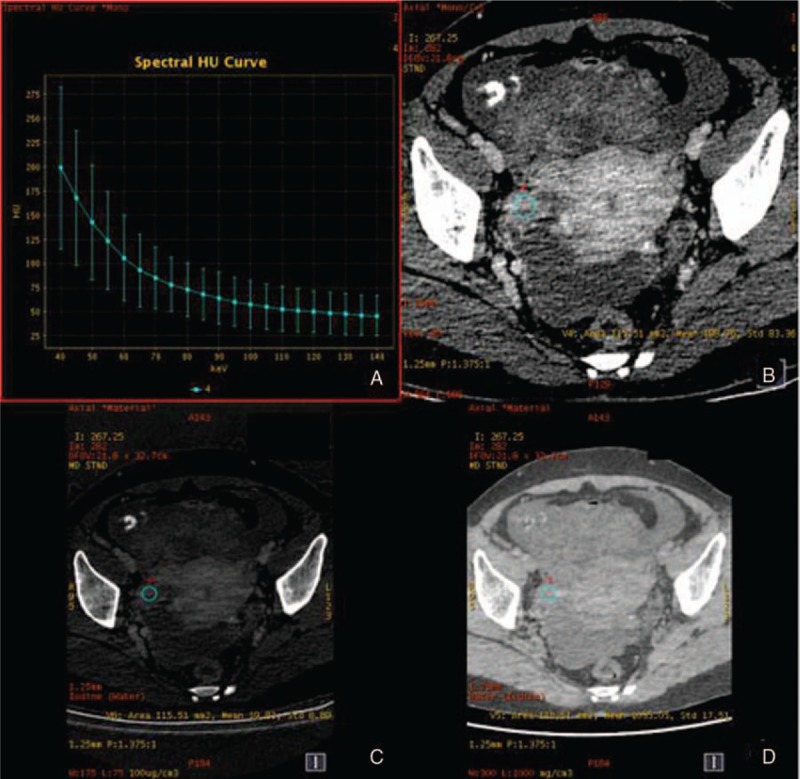
The spectral images of ovarian cancer (the same patient with Fig. 1) in venous phase. A, λ_HU_ = 1.90. B, CT values at 40 keV = 198.76 HU. C, IC 19.83 (100 ug/cm^3^). D, WC 1035.05 mg/cm^3^. λ_HU_ = spectral curve slope, CT = computed tomography, IC = iodine concentration, WC = water concentration.

**Figure 3:**
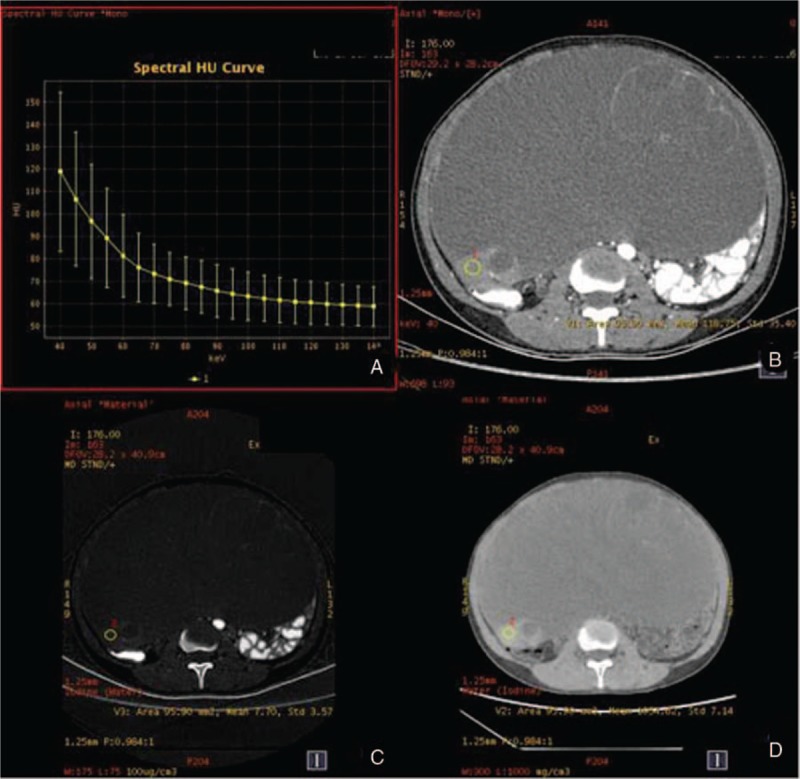
The spectral images of ovarian benign tumor (mucinous cystadenoma) in arterial phase. A, λ_HU_ = 0.92. B, CT values at 40 keV = 118.75 HU. C, IC 7.70 (100 ug/cm^3^). D, WC 1054.62 mg/cm^3^. λ_HU_ = spectral curve slope, CT = computed tomography, IC = iodine concentration, WC = water concentration.

**Figure 4:**
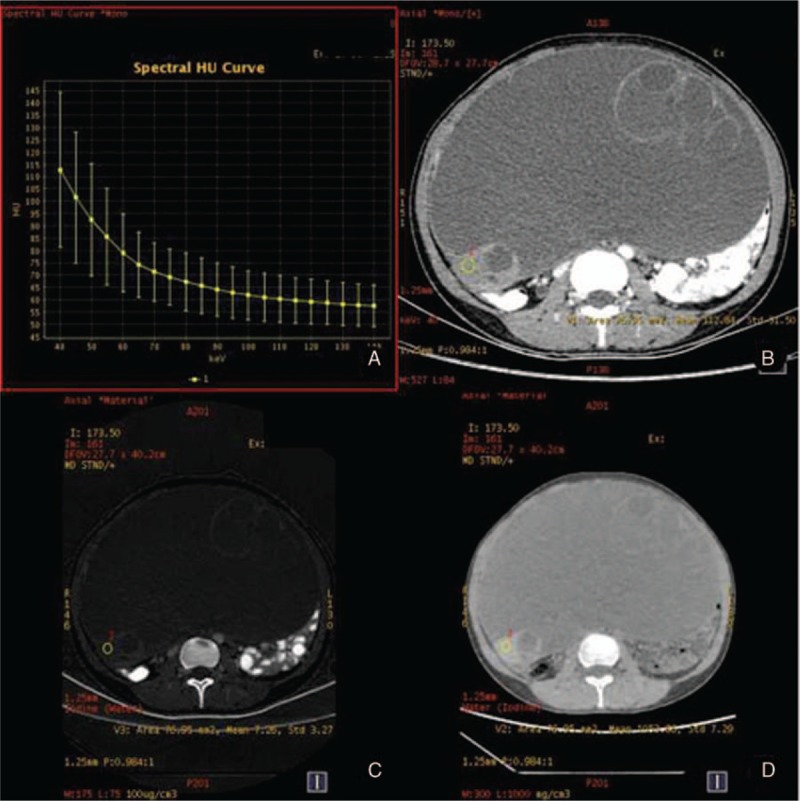
The spectral images of ovarian benign tumor (the same patient with Fig. 3) in venous phase. A, λ_HU_ = 0.85. B, CT values at 40 keV = 112.84 HU. C, IC 7.26 (100 ug/cm^3^). D, WC 1053.66 mg/cm^3^. λ_HU_ = spectral curve slope, CT = computed tomography, IC = iodine concentration, WC = water concentration.

The measurement results of various parameters in the 2 groups are shown in Table 1. There were statistical differences between 2 groups with the parameters of CT values at 40 keV, IC, and λHU (*P* < .05). The results in the ovarian cancer group were higher than those in the benign tumor group, while WC had no statistical difference between these 2 groups (*P* > .05) (Figs. 1–4).

**Table 1:**
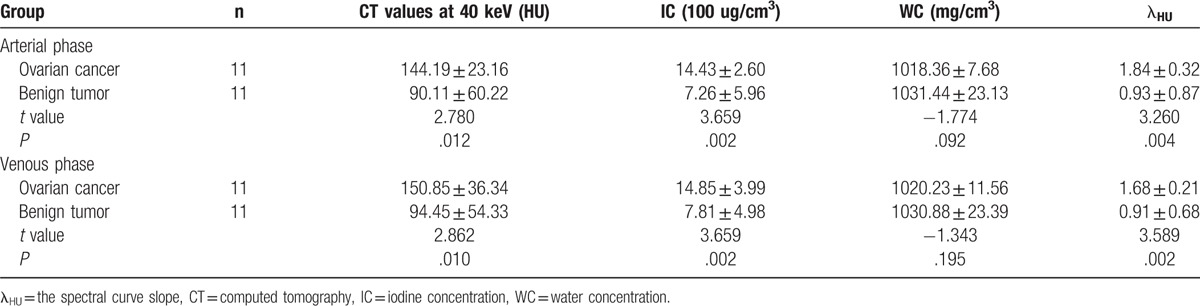
Various parameters of spectral CT in 2 group.

### 3.2 Comparison of the diagnostic effectiveness with various parameters

**Figure 5:**
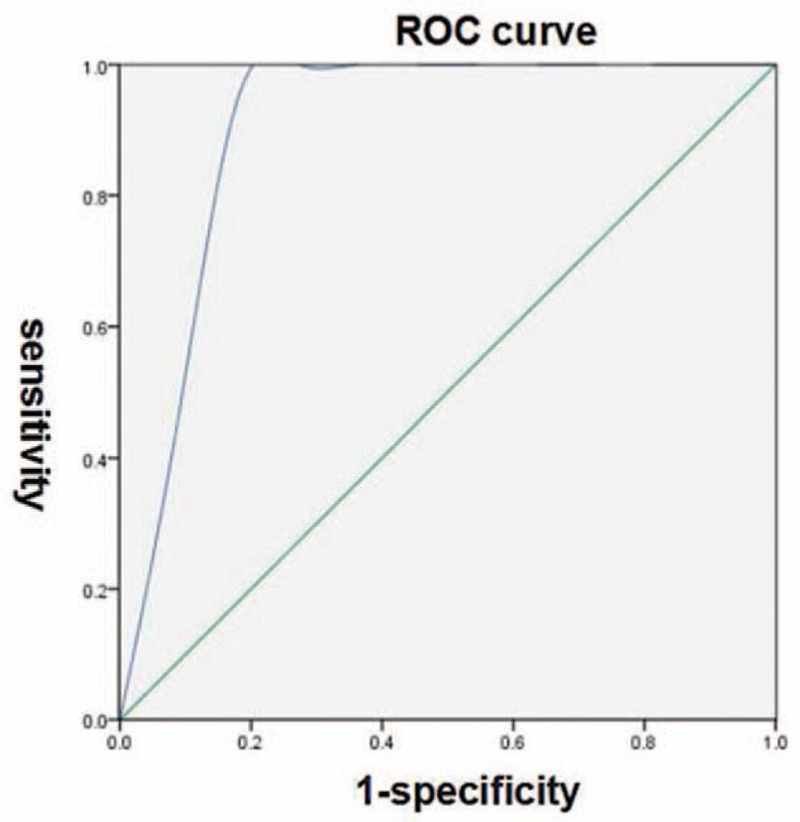
ROC curve with IC in artery phase for the diagnosis of ovarian cancer, AUC = 0.90. AUC = area under ROC curve, IC = iodine concentration, ROC curve = receiver operator characteristic curve.

The results of various parameters are displayed in Table 2. CT values at 40 keV, IC, and λHU had high effectiveness to the diagnosis of ovarian cancer according to the ROC curves. The AUC were more than 0.80 with these 3 parameters in both the arterial and venous phases. The optimal parameter among them was IC in the arterial phase with an AUC of 0.90. Using 10.92 (100 ug/cm^3^) as a threshold value, the sensitivity and specificity for the diagnosis were 88.9% and 94.7% respectively (Fig. 5).

**Table 2:**
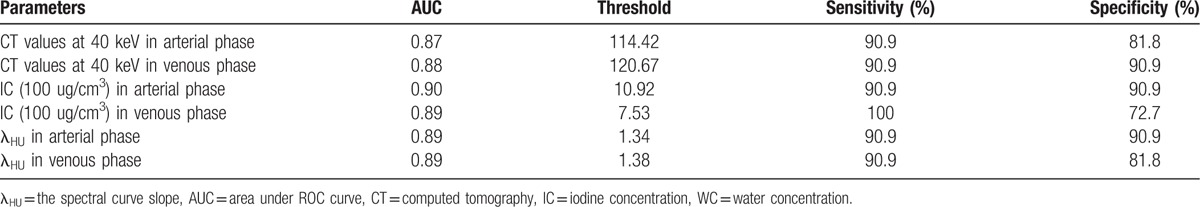
Diagnostic effectiveness with various parameters.

## 4 Discussion

Dual-energy CT is a novel, rapidly emerging imaging technique that offers important new functional and specific information. It is known that CT characterization of body tissue is generally based on differences in X-ray attenuation of different tissues. The spectral CT that we used in our study could momentarily switch between high and low energies (140 kVand 80 kV) in a single X-ray tube in 0.5 ms. This makes material differentiation with monochromatic energy feasible.^[3]^ The monochromatic imaging can help eliminate the ray-hardening artifacts, optimize imaging quality, and increase image contrast.^[4]^ Unlike other dual energy CT techniques, such as 2 X-ray source tubes, a single tube with 2 energy levels for sequential scanning, and a single source with a filter to provide 2 tube voltages, a single tube with high and low energies momentary switching can eliminate movement deviation, so that the image consistency and accuracy can be ensured. With spectral CT, we can get 101 monochromatic images from 40 to 140 keV and derive images for the separation of materials such as calcium, iodine, and water.^[5]^ These provide feasibility for the differentiation of different materials. From spectral CT, we can get multiple parameters, such as CT values at different energies, IC, WC, and effective atomic number. It breaks though the shortcoming of single parameter in routine CT. This technique, maybe, promotes CT development from anatomy to molecular imaging.^[6,7]^

Iodine is the key component in contrast medium during enhanced CT scan. Water is the basic substance in the human body. So iodine and water are the most commonly used base materials for spectral analysis. Iodine concentration can be measured from iodine-based images that can reflect the hemodynamics of tumors objectively. CT attenuation of a special material varies in relation to the energy of the X-ray photons applied. This variation is dependent on the material. Each component has its own spectral curve, and we can make quantitative evaluation by using the spectral curve slope. Now spectral CT has been initially used in many tumors for diagnosis, staging, and prognosis,^[8–12]^ such as liver, pancreas, lung, breast, thyroid, and gastrointestinal tract. However, few studies have paid close attention to the ovarian cancer.

The present data in our study demonstrated that spectral CT with multiple parameters was valuable in the diagnosis of ovarian cancer. There were statistical differences between benign and malignant ovarian tumors with the parameters of CT values at 40 keV, IC, and λHU. These indicated the differences of vascular anatomy and hemodynamics between benign and malignant ovarian tumors. During CT enhancement scanning, the degree of enhancement is dependent on the intravascular iodine content in the tumour, which can reflect the number of supplying vessels and blood flow.^[13,14]^ In malignant tumor, there are rich new vessels, including tortuous vessels and arteriovenous fistula. So the contrast medium can enter into these tumors easily in short time. In contrast, the density of new vessels in benign tumors is relatively low. This results in the slow flow speed of contrast medium entering into the tumors.^[15,16]^ Therefore, the multiple parameters reflecting tumor's hemodynamics, such as CT values at 40 keV, IC, and λHU, maybe exist significant different. During the spectral CT enhanced scan, IC directly reflects the blood perfusion in tumors with positive correlation. In ovarian caners, IC will increase rapidly in the arterial phase. Compared with benign tumors, the changes of IC are more remarkable in the arterial phase. As a result, IC in the arterial phase presented optimal diagnostic effectiveness with an AUC of 0.90. Using 10.92 (100 ug/cm^3^) as a threshold value, the sensitivity and specificity for the diagnosis were 88.9% and 94.7% respectively.

There are several limitations to the present study. First, the number of patient cases used in this study was too few to properly classify ovarian tumors in comparison with different pathological types and grading. And for the cancer group, it included 3 kinds of primary tumors and 2 metastatic tumors. Further studies should be continued in the future with a large sample to reduce the deviation. Second, further studies should be carried out for some special ovarian tumors. For example, there was 1 case of sclerosing stromal tumor mimicking endodermal sinus tumor. Because the onset age and enhancement patterns in these 2 types of tumors were similar. CT values at 40 keV, IC, and λHU were all high. It was difficult to differentiate these 2 types of tumors by using these targets above. Although it was a particular case, it still indicated the limitation of our study. In the future, we will perfect the targets to improve the diagnosis accuracy.

In conclusion, spectral CT with multiple parameters can partly reflect the hemodynamics of ovarian tumors. As a supplementary imaging technology, spectral CT can preliminarily differentiate benign and malignant ovarian tumors.
